# 

**Published:** 1886-12

**Authors:** 


| | Page |
|---|---|
| ORIGINAL COMMUNICATIONS: | |
| A Case of Actinomycosis. A. J. Ochsner | 1 |
| The Relation of State Medicine to Architecture. O. C. DeWolf | 4 |
| Stricture of the Rectum. A. E. Hoadley | 18 |
| A Case of Hysteric Trance | 28 |
| Ichthyol in Skin Diseases. J. Zeisler | 32 |
| Prophylaxis in Rhinitis Sympathetica. S. O. Richey | 42 |
| EDITORIAL: | |
| Pasteurism | 46 |
| Tubercle Bacillus | 48 |
| Zonular Cataract and Dental Malformations | 49 |
| SOCIETY PROCEEDINGS: | |
| *Chicago Medical Society; Meeting November 1* | 52 |
| *Chicago Society of Ophthalmology and Otology; Meeting June 8* | 60 |
| Congenital Ectopia Lentis. H. M. Starkey | 60 |
| Ligature for Eutropion of the lower Eye-lid. W. T. Montgomery | 61 |
| *Meeting October 12.* | |
| The Rational Treatment of Patients after Cataract Operations. F. C. Hotz | 65 |
| ABSTRACTS: | |
| Onanism in Young Children. Professor Hirschsprung | 68 |
| Cocaine Intoxication. Dr. Commanus | 74 |
| A Peculiar Form of Motor Disturbance of the Pupil. J. Salgó | 76 |
| BOOK REVIEWS: | |
| Local Anæsthesia in General Medicine and Surgery. J. L. Corning | 77 |
| A Manual of Surgery. F. Treves | 79 |
| A Treatise on the Practice of Medicine. R. Bartholow | 80 |
| The Healing of Arteries after Ligature in Man and Animals. J. C. Warren | 81 |
| A Manual of Differential Medical Diagnosis. C. W. Cutler | 83 |
| Lectures on Dietetics and Dyspepsia. W. Roberts | 84 |
| Dictionary of Practical Surgery | 84 |
| Massage as a Mode of Treatment | 85 |
| A Manual of Minor Surgery and Bandaging. C. Heath | 86 |
| A Treatise on the Principles and Practice of Medicine. A. Flint | 87 |
| An Atlas of Clinical Microscopy | 89 |
| Books Received | 90 |

